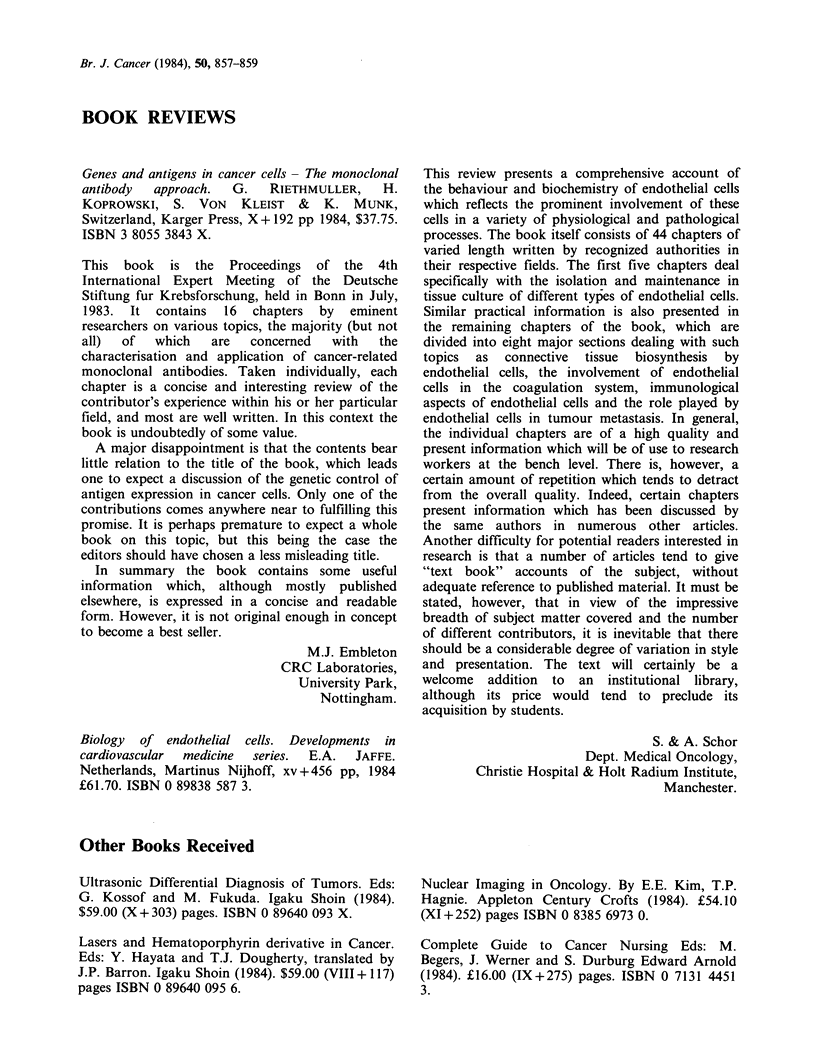# Biology of endothelial cells. Developments in cardiovascular medicine series

**Published:** 1984-12

**Authors:** S.A. Schor


*Biology of endothelial cells. Developments in cardiovascular medicine series.* E.A. JAFFE. Netherlands, Martinus Nijhoff, xv+456 pp, 1984 £61.70. ISBN 0 89838 587 3.

This review presents a comprehensive account of the behaviour and biochemistry of endothelial cells which reflects the prominent involvement of these cells in a variety of physiological and pathological processes. The book itself consists of 44 chapters of varied length written by recognized authorities in their respective fields. The first five chapters deal specifically with the isolation and maintenance in tissue culture of different types of endothelial cells. Similar practical information is also presented in the remaining chapters of the book, which are divided into eight major sections dealing with such topics as connective tissue biosynthesis by endothelial cells, the involvement of endothelial cells in the coagulation system, immunological aspects of endothelial cells and the role played by endothelial cells in tumour metastasis. In general, the individual chapters are of a high quality and present information which will be of use to research workers at the bench level. There is, however, a certain amount of repetition which tends to detract from the overall quality. Indeed, certain chapters present information which has been discussed by the same authors in numerous other articles. Another difficulty for potential readers interested in research is that a number of articles tend to give "text book" accounts of the subject, without adequate reference to published material. It must be stated, however, that in view of the impressive breadth of subject matter covered and the number of different contributors, it is inevitable that there should be a considerable degree of variation in style and presentation. The text will certainly be a welcome addition to an institutional library, although its price would tend to preclude its acquisition by students.

S. & A. Schor
Dept. Medical Oncology,
Christie Hospital & Holt Radium Institute,
Manchester.